# Four consecutive yearly point-prevalence studies in Wales indicate lack of improvement in sepsis care on the wards

**DOI:** 10.1038/s41598-021-95648-6

**Published:** 2021-08-10

**Authors:** Maja Kopczynska, Harry Unwin, Richard J. Pugh, Ben Sharif, Thomas Chandy, Daniel J. Davies, Matthew E. Shield, David E. Purchase, Samuel C. Tilley, Arwel Poacher, Lewis Oliva, Sam Willis, Isabelle E. Ray, John Ng C. Hui, Bethany C. Payne, Eilis F. Wardle, Fiona Andrew, Hei Man Priscilla Chan, Jack Barrington, Jay Hale, Joanna Hawkins, Jess K. Nicholas, Lara E. Wirt, Lowri H. Thomas, Megan Walker, Myat P. Pan, Tallulah Ray, Umair H. Asim, Victoria Maidman, Zeid Atiyah, Zain M. Nasser, Zhao Xuan Tan, Laura J. P. Tan, Tamas Szakmany, The Welsh Digital Data Collection Platform collaborators, Maria Hobrok, Moriah Thomas, Annie Burden, Nadia Youssef, Katherine Carnegie, Helena Colling-Sylvester, Natasha Logier, Meshari Alsaeed, Hannah Williams, Arfa Ayob, Nor Farzana, Sweta Parida, David Lawson, Emily Evans, Laura Jane Davis, Billie Atkins, Llywela Wyn Davies, Lee Sanders-Crook, Steffan Treharne Seal, Alice Cains, Katy Crisp, Sarah Venning, Ella Sykes, Stephanie Narine, Georgia Parry, Emily Angela Dillon, Qi Zhuang Siah, Ting Yang, Tyler Jones, Parvathi Thara, Emma Wood, Georgina St Pier, Richard Betts, Kyriaki Mitsaki, Mari Tachweed Pierce, Sioned Davies, Yakeen Hafouda, Erin Ifan, Grace Lacey, Francesca Mitchell, John Lynch, Michal Mazur, Lezia D’Souza, Bethan Ponting, Terrance Lau, Ruairidh Kerrigan, Lucy Morgan, Roshan Vindla, Claudia Zeicu, Becky James, Amirah Amin Ariff, Wan Binti Wan Azzlan, Charlotte Collins, Elizabeth Wickens, Alisa Norbee, Aliya Zulkefli, Thomas Haddock, Megan Thomas, Matthew Lee, Miriam Cynan, Nik-Syakirah Nik Azis, Imogen Hay, Catherine Russell, Margriet Vreugdenhil, Mustafa Abdimalik, Joseph Davies, Peter Havalda, Angharad Evans, Kate Robertson, Grace Gitau, Mei-yin Gruber, Thomas Telford, Anas Qarout, Naomi Nandra, Hannah Garrard, James Cutler, Rhiannon Tammy Jones, Amy Prideaux, Timothy Spence, Sarah Hardie, Harriet Seymour, Matthew Warlow, Shanali Thanthilla, Thomas Downs, Nina Foley, Chad McKeown, Akshita Dandawate, Holleh Shayan-Arani, Ellie Taylor, Oliver Kyriakides, Rachel Price, Ffion Haf Mackey, Emily Haines, Samuel Chun, Nilarnti Vignarajah, Tessa Chamberlain, Dongying Zhao, Nayanatara Nadeesha T. Tantirige, Naomi Dennehey, Georgina Evans, John Watts, Ceri Battle, Ryan Jones, Selina Jones, Charlotte James, James O’Hanlon, Isabella Bridges, Bethany Hughes, Leo Polchar, Elise Bisson, Charlotte Mykura, Lara Money, Joshua McKenna, Sarah Kinsman, Demiana Hanna, Emily Baker, Harrison Sprague, Liam Sharma, Tom Pontin, Emma Shore, Tamara Hughes, Sam Nightingale, Philby Baby, Matthew Shield, Alice Cross, Jenna Boss, Olivia Ross, George Ashton, Kimaya Pandit, Daniel Davies, Cameron Garbutt, Charlotte Johnston, Marcus Cox, Chantal Roberts, Alessia Waller, Laura Heekin, Kathy Wang, Rhianna Church, Shrina Patel, Marianne Broderick, Hannah Whillis, Daniel Craig Hathaway, Emel Yildirim, Caitlin Atkins, Elin Walters, Carys Durie, Robert James Hamilton Sinnerton, Benjamin Tanner, Julimar Abreu, Kiran Bashir, Vincent Hamlyn, Amelia Tee, Zoe Ann Hinchcliffe, Rita Otto, Georgie Covell, Megan Stone, Katherine Godfray, Rhidian Caradine, Hannah Beetham, Adanna Nicole Anomneze-Collins, Jeanette Tan, Yasmina Abdelrazik, Azizah Khan, Nabihah Malik, Aidan Clack, Tyler Thomas, Adam George Mounce, Anoopama Ramjeeawon, Ndaba Mtunzi, Duncan Soppitt, Jack Wellington, Robert Buchanan Ross, Danielle Lis, Rebecca Parsonson, Jude Joseph-Gubral, Ajitha Arunthavarajah, Aaron Harris, Henry Atkinson, Jessica Webster, Tim Burnett, Josephine Raffan Gowar, Sam DeFriend, Jasmine Whitaker, Elizabeth Beasant, Luis Macchiavello, Danyal Usman, Abdullah Mahdi, Tiffany Ye Tze Shan, Nick Savill, Jennifer Gee, Lizzie Hodges, Ami Desai, Hannah Rossiter, Matthew Taylor, Kevin Pinto, Eleanor Hartley, Oscar Emanuel, Rhiannon Long, Megan Selby, Alexandra Urquhart, Matthew Ashman, Elizabeth Adcock, Amelia Dickinson, Rebecca Jordache, Rym Chafai El Alaoui, Sophie Stovold, Sam Vickery, Nia Jones, Alice O’Donnell, Monty Cuthbert, Osa Eghosa, Muhammad Karim, Lowri Williams, Louise Tucker, Tom Downs, Rebecca Walford, Annabelle Hook, Adam Mounce, Emily Eccles, Ross Edwards, Kirtika Ramesh, Charlie Hall, Maria Lazarou, Rhidian Jones, Katy McGillian, Hari Singh Bhachoo, Zoe Teh, Vithusha Inpahas, Ruchi Desai, Yusuf Cheema, Andrew Hughes, Olivia Cranage, Felicity Bee, Khalid Osman, Humza Khan, Jennifer Pitt, Charlotte Pickwick, Jorge Carter, Fiona Andrew, Naseera Seedat, Roshni Patel, Alicia Boam, Jessica Randall, Beth Bowyer, Josh Edwards, Natasha Jones, Emma Walker, Ailsa MacNaught, Swagath Balachandran, Abbie Shipley, Jennifer Louise Kent, Bethany Davies, Emma Withers, Krishna Parmar, Lucie Webber, Angelica Sharma, Amy Handley, Alexandra Gordon, Lucy Allen, Rebecca Paddock, Harriet Penney, Lopa Banerjee, Chloe Victoria Vanderpump, Kate Harding, John Burke, Orsolya Minik, Nia Jarrett, Ellie Rowe, Adanna Anomneze-Collins, Harry Griffiths, Sarah Pengelly, Ffion Bennett, Ahmed Bilal, Abdullah El-badawey, Bethan Ellis, Luke Cook, Harriet Elizabeth Valentine Maine, Kiri Armstrong, Hannah Beresford, Timia Raven-Gregg, Tom Liddell-Lowe, Caitlin Ong, Harriet Reed, Frederika Alice St John, Weronika Julia Kozuch, Irukshi Anuprabha Silva, Sin Ting Natalie Cheng, Umme-Laila Ali, Noreena Syed, Luke Murphy, Thomas Grother, Harry Smith, Rachel Watson, Omar Marei, Emma Kirby, Anna Gilfedder, Lydia Maw, Sarah O’Connor, Charlotte Maden, Helena Jones, Hazel Preston, Nur Amirah Binti Maliki, Mark Zimmerman, Jessica Webber, Llewelyn Jones, Rebecca Phillips, Lauren McCarthy, Emily Hubbard, Leo Duffy, Abigail Guerrier Sadler, Owen Richards, Charles King, Charlotte Killick, Yusuf Chema, Kavita Shergill, Yi Huen Lillian Lau, Hannah Mustafa Ali, Lucas Wilcock, Molly Timlin, Ayeesha Rela, Daniel Smith, Sarah Ireland, Jennifer Evans, Nayanatara Poobalan, Jessica Pearce, Thivya V. Vadiveloo, Zoe Black, Daniel Elis Samuel, Humaira Hussain, Rebecca Creamer, Maham Zafar, Ahmad Almazeedi, Hannah Brunnock, Mekha Jeyanthi, Poorya Moghbel, Katie Kwan, Isobel Sutherland, Frank Davis, Abigail Rogers, Clare Chantrill, Amal Robertson, Jonathan Foulkes, Rahana Khanam, Jomcy John, Sarah Hannah Meehan, Huria Metezai, Hannah Dawson, Navrhinaa Vadivale, Camilla Lee, Amrit Dhadda, Sian Cleaver, Genna Logue, Joy Inns, Isabel Jones, Robyn Howcroft, Carys Gilbert, Matthew Bradley, Louise Pike, Rachel Keeling, Charldré Banks, Eleanor Cochrane, James McFadyen, Matthew Mo, Emily Ireland, Esme Brittain, Ihssen Laid, Charlotte Green, Adriel Mcforrester, Tu Xuong Michelle Ly, Mariana Nalbanti, Raven Joseph, Jack Tagg, Ayako Niina, Tyler Joshua Jones, Natalie Hoyle, Patrick Benc, Ellen Davies, Meng-Chieh Wu, David Fellows, Eloise Baxendale, Karishma Khan, Andrew Forrester, Oliver Moore, Hse Juinn Lim, Aimee Owen, Faris Hussain, Nima-banu Allybocus, Maneha Sethi, Harry Waring, Adeel Khan, Claire Smith, Nicholas Doyle, Mohammad Yahya Amjad, Luke Galloway, Paul Morgan, Gemma Ellis, Robert Lundin, Haamed Al Hassan, Bethan Markall, Namratha Kaur, Emmanuel Onyango, Heather Beard, Elliot Field, Ellen Nelson-Rowe, Lizzie Adcock, Amelia Stoddart, Frederika St John, Mathoorika Sivananthan, Rhys Jones, Sung Yeon Kwak, Lily Farakish, Holly Rhys-Ellis, Kate Moss, Tessa David, Talea Roberts, Annie Quy, Aniket Paranjape, Felicity Bee, Nutchanun Poolworaluk, Mary Keast, Si Liang Yao, Dion Manning, Isobel Irwin, Emelia Boggon, Ibrahim Alkurd, Genevieve Lawerece, Jade Brown, Emily Murphy, Evie Lambert, Jeremy Guilford, Mariam Almulaifi, Sashiananthan Ganesananthan, Berenice Cunningham-Walker, Chloe Spooner, Akanksha Kiran, Nabeegh Nadeem, Vidhi Unadkat, Esme Sparey, David Li, Jessica Smith, India Corrin, Amit Kurani, Paul McNulty, Ceri Brown, Wojciech Groblewski, Szilvia Szoke, Amelia Redman, Esther McKeag, Anastasia Donnir, Gaautham Ravishangar, Emanuela Howard, Charlotte Salmon, Sara Tanatova, Jasmine Kew, Megan Eilis Clark, Ellen Hannay, Olesya Godsafe, Christina Houghton, Francesca Lavric, Rachel Mallinson, Chris Littler, Harsha Reddy, Andrew Campbell, Benedict Soo, Rachel Evans, Georgina Donowho, Alexandra Cawthra, Maddison Davies, Matthew Lawrence Ashman, Jamie Scriven, James Vautrey, Shannon Seet, Imogen Britton, Abigail Hodgson, Emma Twohey, Joseph Robbins, Vanessa Yeo Yung Ling, Kimiya Asjadi, Carven Chin Yee Shean, Zoe McCarroll, Oritseweyimi Amatotsero, Antonia Ashaye, Josephine Acheampong, Ayowade Adeleye, Saber Ahmed, Alexandra Chrysostomou, Eshen Ang, Niamh McSwiney, Yin Yin Lim, Zong Xuan Lee, Svetlana Kulikouskaya, Nur Zulkifili, Sheryl Lim, Lim Xin, Adiya Urazbayeva, Nur Haslina Ahmad Hanif, Yau Ke Ying, Alice Coleclough, Eilis Higgins, Naomi Spencer, Tze Gee Ng, Sam Booth, Stephanie Wai Yee Ng, Christian P. Subbe, Isabella Patterson, Wen Li Chia, Abdullah Mukit, Hei Yi Vivian Pak, Felicity Lock, Mariana Nalmpanti, Shôn Alun Thomas, Tanisha Burgher, Alfred Wei Zhen Yeo, Siwan Powell Jones, Charlie Miles, Millicent Perry, Holly Burton, Katharine Powell, Luthfun Nessa, Aalaa Fadlalla, Rhian Morgan, Elizabeth Hodges, Amelia Heal, Chloe Scott, Alice Tayler, Abduahad Taufik, James Cochrane, Sieh Yen Heng, Alex Cooper, Henrik Graf von der Pahlen, Isabella Talbot, Robin Gwyn Roberts, Jessica Sharma Smith, Aisling Sweeney, Cerian Roberts, Laura Bausor, Chania Lambirnudi, Daniah Thomas, Elen Wyn Puw, Ronan A. Lyons, Judith E. Hall

**Affiliations:** 1 Salford Royal NHS Foundation Trust, Manchester, UK; 2 Cardiff University School of Medicine, Cardiff, UK; 3 Intensive Care Medicine Glan Clwyd Hospital, Betsi Cadwaladr University Health Board, Bodelwyddan, UK; 4 Prince Charles Hospital, Cwm Taf Morgannwg University Health Board, Merthyr Tydfil, UK; 5 The Grange University Hospital, Aneurin Bevan University Health Board, Cwmbran, Wales UK; 6 College of Medicine, Swansea University Medical School, Swansea, UK; 7 Cardiff and Vale University Health Board, University Hospital of Wales, Cardiff, UK; 8 Glangwili General Hospital, Hywel Dda University Health Board, Carmarthen, UK; 9 Prince Charles Hospital, Cwm Taf Morgannwg University Health Board, Merthyr Tydfil, UK; 10 Wrexham Maelor Hospital, Betsi Cadwaladr University Health Board, Wrexham, UK; 11 West Suffolk NHS Foundation Trust, Bury St Edmunds, Suffolk, UK; 12 University Hospital of Wales, Cardiff and Vale University Health Board, Cardiff, UK; 13 Department of Anaesthesia, Intensive Care and Pain Medicine, Division of Population Medicine, Cardiff University, Cardiff, UK; 14 Intensive Care Medicine, Critical Care Directorate, Grange University Hospital, Aneurin Bevan University Health Board, Cwmbran, UK; 15 Bronglais General Hospital, Hywel Dda University Health Board, Aberystwth, UK; 16 Morriston Hospityal, Swansea Bay University Health Board, Swansea, UK; 17 Nevil Hall Hospital, Aneurin Bevan University Health Board, Abergavenny, UK; 18 Princess of Wales Hospital, Cwm Taf Morgannwg University Health Board, Bridgend, UK; 19 Royal Glamorgan Hospital, Cwm Taf Morgannwg University Health Board, Llantrisant, UK; 20 Royal Gwent Hospital, Aneurin Bevan University Health Board, Newport, UK; 21 University Hospital Llandough, Cardiff and Vale University Health Board, Cardiff, UK; 22 Withybush General Hospital, Hywel Dda University Health Board, Haverfordwest, UK; 23 Ysbyty Gwynedd, Betsi Cadwaladr University Health Board, Bangor, UK; 24 Health Data Research UK, Swansea University, Swansea, UK

**Keywords:** Bacterial infection, Epidemiology, Fever, Antimicrobial therapy

## Abstract

The ‘Sepsis Six’ bundle was promoted as a deliverable tool outside of the critical care settings, but there is very little data available on the progress and change of sepsis care outside the critical care environment in the UK. Our aim was to compare the yearly prevalence, outcome and the Sepsis Six bundle compliance in patients at risk of mortality from sepsis in non-intensive care environments. Patients with a National Early Warning Score (NEWS) of 3 or above and suspected or proven infection were enrolled into four yearly 24-h point prevalence studies, carried out in fourteen hospitals across Wales from 2016 to 2019. We followed up patients to 30 days between 2016–2019 and to 90 days between 2017 and 2019. Out of the 26,947 patients screened 1651 fulfilled inclusion criteria and were recruited. The full ‘Sepsis Six’ care bundle was completed on 223 (14.0%) occasions, with no significant difference between the years. On 190 (11.5%) occasions none of the bundle elements were completed. There was no significant correlation between bundle element compliance, NEWS or year of study. One hundred and seventy (10.7%) patients were seen by critical care outreach; the ‘Sepsis Six’ bundle was completed significantly more often in this group (54/170, 32.0%) than for patients who were not reviewed by critical care outreach (168/1385, 11.6%; *p* < 0.0001). Overall survival to 30 days was 81.7% (1349/1651), with a mean survival time of 26.5 days (95% CI 26.1–26.9) with no difference between each year of study. 90-day survival for years 2017–2019 was 74.7% (949/1271), with no difference between the years. In multivariate regression we identified older age, heart failure, recent chemotherapy, higher frailty score and do not attempt cardiopulmonary resuscitation orders as significantly associated with increased 30-day mortality. Our data suggests that despite efforts to increase sepsis awareness within the NHS, there is poor compliance with the sepsis care bundles and no change in the high mortality over the study period. Further research is needed to determine which time-sensitive ward-based interventions can reduce mortality in patients with sepsis and how can these results be embedded to routine clinical practice.

*Trial registration* Defining Sepsis on the Wards ISRCTN 86502304 https://doi.org/10.1186/ISRCTN86502304 prospectively registered 09/05/2016.

## Introduction

Sepsis is defined as dysregulated host response to infection with sequential organ failure. It is a complex disorder and is associated with high mortality^1^. Despite increased awareness, sepsis remains a major challenge and economic burden to healthcare globally^2–5^. To improve patient mortality, sepsis requires early recognition and urgent treatment^6^. Previously much attention was dedicated to the identification and treatment of patients at risk of poor outcomes within intensive care units (ICU)^7,8^. However, it is now known that the majority of patients with sepsis present in the emergency department (ED) and on general wards, with associated high mortality^9–11^.

Since the inception of the sepsis resuscitation bundle by the Surviving Sepsis Campaign (SSC) over a decade ago, completion rates have been reportedly low^12–14^. As the initial SSC bundle was heavily reliant on complex interventions, typically performed in a critical care environment, the ‘Sepsis Six’ bundle was promoted as a more deliverable tool outside of the critical care settings^15^. Although high-profile cases and systematic campaign from advocacy groups helped to increase awareness of the condition in the last decade, there is very little data available on the progress and change of sepsis care outside the critical care environment in the UK^15,16^. While the use of sepsis screening tools and the delivery of the ‘Sepsis Six’ bundle is now a key performance indicator in many institutions, external scrutiny of such initiatives is lacking^17,18^. The aim of our study was to examine the changes in care processes and outcomes over a four-year period, by utilising our yearly All Wales point-prevalence study on sepsis.

## Methods

### Study design and participants

We performed a secondary analysis on the patient populations recruited into four annual multi-centre 24-h point-prevalence studies conducted on the third Wednesday of October from 2016 to 2019. The study was conducted accordance with relevant guidelines and regulations including the Declaration of Helsinki. The Defining Sepsis on the Wards project was prospectively registered with an international trial registry (ISRCTN86502304).

Patients were recruited from each of the 14 acute hospitals across Wales, all of which had 24-h consultant cover in the ED and non-selective intake. Participating hospitals were identified through local collaborators via the Welsh Intensive Care Society Audit and Research Group. We screened all patients presenting to the ED and on the general wards. At the start of the study days at 08:00, data collectors systematically screened every patient on the acute in-patient wards within 4 h, then continued screening for any potential new participants until 07:59 the next morning. In each hospitals dedicated data collectors were stationed in the ED during the 24 h periods. We approached all patients with NEWS ≥ 3 in whom the treating clinical teams had a high degree of clinical suspicion of an infection (documented as such in the medical or nursing notes), and following the patients or their proxy, in cases of patients lacking capacity, gave written informed consent and were recruited to the study. Patients under 18 and those cared for in critical care or mental health units were excluded.

Local investigators were identified and were supported by three national coordinators. Key study information was provided through e-mails, face-to-face training and online video tutorials, which included the protocol, answers to key questions and description of the electronic case report form (eCRF). The details of the digital data collection platform developed for this study have been published previously^19^. Medical students working in pairs to ensure data validity and appropriate clinical knowledge, acted as data collectors, using tablets for electronic data collection and transfer. The tablets contained all supporting information needed for the study, including national formulary. Data collectors were supported by continuous online web-chat, which made the senior clinicians and the medical student national coordinators available throughout the study period. We referred patients to the clinical teams if the medical student data collectors felt they needed urgent medical attention due to their condition, in line with the requirements of the ethics approval. To facilitate linkage to national databases for the collection of follow-up data, we collected patient-identifiable data and entered it on to the secure data collection tool^19^. Further description of the methodology and performance of this platform is outlined in previous publications^16,18–23^.

We collected data from medical and nursing records on pre-admission patient characteristics, co-morbidities, physiological and laboratory values, Dalhousie clinical frailty score, and management actions such as the completion of the ‘Sepsis Six’ bundle and involvement of critical care outreach. In 2016, we conducted follow-up data collection for our primary outcome of all-cause mortality at 30 days from enrolment. In subsequent years (2017–2019) we conducted follow-up at 30 and 90 days.

Policy content: During the study period all of the participating hospitals were actively engaged in the Rapid Response to Acute Illness Learning Set (RRAILS) programme led by 1000 Lives Improvement. In 2013, all hospitals in Wales implemented the use of NEWS, with a score of six or above set to trigger the escalation of patients to senior decision makers or for consideration of referral to critical care outreach. RRAILS promoted the use of standardised sepsis screening tool across the hospital since 2008 (see Supplementary Figure 1). In 2018 the Welsh Government introduced a quality improvement performance indicator for the completion of ‘Sepsis Six’ in all acute hospitals based on the RRAILS tool.

### Statistical analysis

Categorical variables are described as proportions and are compared using Chi square test. Continuous variables are described as median and interquartile range (IQR) and compared using Mann–Whitney U test. We plotted Kaplan–Meier survival curves and compared time-to-event data using log-rank testing. The starting point for the survival analysis was the data collection day. We estimated the respective hazard ratios (HRs) for the primary outcome within 30 days with a Cox proportional hazards model after adjustment for measured confounders. The model fit was assessed by the − 2 log likelihood statistics and Chi‐square test.

To increase sample size and to enable the inclusion of patients from all four study years, the primary analysis was performed on 30-day follow up results only. However, we also performed a subgroup analysis using the 90-day survival data using the results from the 2017 to 2019 studies. A two-tailed *p*-value < 0.05 was considered statistically significant. All statistical tests were calculated using SPSS 25.0 (SPSS Inc., Chicago, IL). Data visualisation was performed in R (Version 1.2.1335) with packages: ggplot2 (v3.3.3), dplyr (v1.0.5), UpSetR (v1.4.0), ComplexHeatmap (v2.7.8.1000) and sunburstR (v2.1.5), utilising repositories from Github (hms-dbmi/UpSetR, jokergoo/ComplexHeatmap and timelyportfolio/sunburstR)^24,25^.

### Ethical approval and consent to participate

Ethical approval was granted by the South Wales Regional Ethics Committee (16/WA/0071, 15/04/2016) and patients or legal representatives gave written informed consent.

## Results

### Patient characteristics

**Figure 1:**
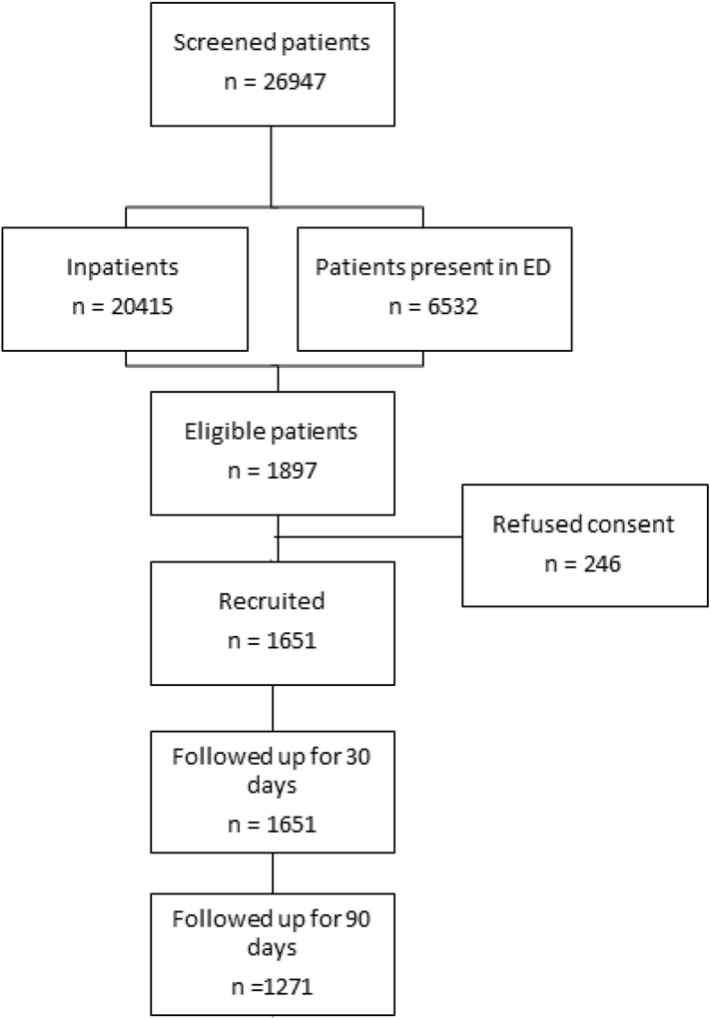
Study flow diagram and eventual study sample. ED; emergency department.

Over the four annual 24-h point-prevalence study periods, we screened a total of 26,947 patients, of whom 1651 met inclusion criteria and were subsequently recruited (Fig. 1).

Patient demographics and clinical characteristics for each year of study are shown in Table 1. The median age (IQR [range]) of participants was 73 years (60–82 [18–103]) and more females 852 (51.6%) than males 799 (48.4%) were recruited. The median (IQR) frailty score was 5 (3–6). Age, gender, and frailty of participants did not vary between years (Table 1).

**Table 1:** Demographics, clinical characteristics and survival of patients in each year of study. Values are median (IQR [range]), number (proportion) or mean (95%CI).

	Year					
	2016 (n = 380)	2017 (n = 459)	2018 (n = 413)	2019 (n = 399)	All years (n = 1651)	*P* value
**Patient demographics**						
Age: median years	74 (61–83 [18–100])	73 (62–84 [18–103])	73 (59–81 [19–99])	73 (60–81 [19–99])	73 (60–82 [18–103])	0.41
Sex: male	180 (47.4%)	231 (50.3%)	213 (51.6%)	175 (43.9%)	799 (48.4%)	0.12
Survival to 30 days	380 (79.5%)	372 (81.0%)	343 (83.1%)	332 (83.2%)	1349 (81.7%)	0.38
Mean survival in 30-day follow-up (days)	25.5 (24.5–26.4)	26.6 (25.8–27.3)	26.8 (26.0–27.6)	26.9 (26.1–27.6–)	26.5 (26.1–26.9)	0.39
**Clinical characteristics**						
COPD	112 (30.9%)	118 (26.2%)	117 (30.1%)	135 (34.8%)	482 (30.3%)	0.06
Diabetes	75 (20.7%)	98 (21.8%)	89 (22.9%)	71 (18.3%)	333 (20.9%)	0.44
Drugs of abuse	5 (1.4%)	8 (1.8%)	11 (2.8%)	7 (1.8%)	31 (1.9%)	0.51
Heart failure	45 (12.4%)	49 (10.9%)	50 (12.9%)	39 (10.1%)	183 (11.5%)	0.58
Hypertension	107 (29.5%)	165 (36.7%)	145 (37.3%)	140 (36.1%)	557 (35.0%)	0.09
Ischemic heart disease	63 (17.4%)	82 (18.2%)	65 (16.7%)	67 (17.3%)	277 (17.4%)	0.95
Liver disease	11 (3.0%)	13 (2.9%)	19 (4.9%)	16 (4.1%)	59 (3.7%)	0.39
Neuromuscular	13 (3.6%)	16 (3.6%)	11 (2.8%)	12 (3.1%)	52 (3.3%)	0.92
Recent chemotherapy	14 (3.9%)	21 (4.7%)	15 (3.9%)	24 (6.2%)	74 (4.7%)	0.37
Frailty score: median*	5 (3–6)	5 (3–6)	4 (3–6)	5 (3–6)	5 (3–6)	0.26
DNA-CPR	90 (24.1%)	123 (27.5%)	92 (24.5%)	109 (27.9%)	414 (26.1%)	0.49
NEWS ≥ 6	115 (30.3%)	130 (28.3%)	120 (29.1%)	121 (30.3%)	486 (29.4%)	0.90

*Frailty score range was from 1 (“very fit”) to 9 (“terminally ill”) in all years.

Data was missing for frailty score for a total of 64 patients; 7 in 2016, 12 in 2017, 37 in 2018 and 8 in 2019.

COPD, Chronic Obstructive Pulmonary Disease, DNA-CPR, Do Not Attempt Cardiopulmonary Resuscitation order, NEWS, National Early Warning Score, IQR, interquartile range, 95%CI, 95% confidence interval.

### Sepsis management

Overall, 289 (18.2%) patients were screened for sepsis using the ‘All Wales sepsis screening tool’. The ‘Sepsis Six’ bundle was completed on 223 (14.0%) occasions. There were no significant trends in completion rates of the screening tools between 2016 and 2019, nor in the proportion of patients seen by critical care outreach (Table 2).

**Table 2:** Screening and management of patients in each year of study. Values are number (proportion).

	Year					
	2016 (n = 373)	2017 (n = 446)	2018 (n = 380)	2019 (n = 391)	All years (n = 1590)	*P* value
Completed ‘Sepsis Six’ bundle	44 (11.8%)	63 (14.1%)	58 (15.3%)	58 (14.8%)	223 (14.0%)	0.53
Completed All Wales screening tool	59 (15.8%)	100 (22.4%)	62 (16.5%)	68 (17.4%)	289 (18.2%)	0.06
Number of patients seen by critical care outreach	33 (8.8%)	56 (12.6%)	32 (8.6%)	49 (12.5%)	170 (10.7%)	0.11

Data was missing for; Completed All Wales Screening tool for 4 patients in 2018; Data was also missing for number of patients seen by critical care outreach for 6 patients in 2018.

**Figure 2:**
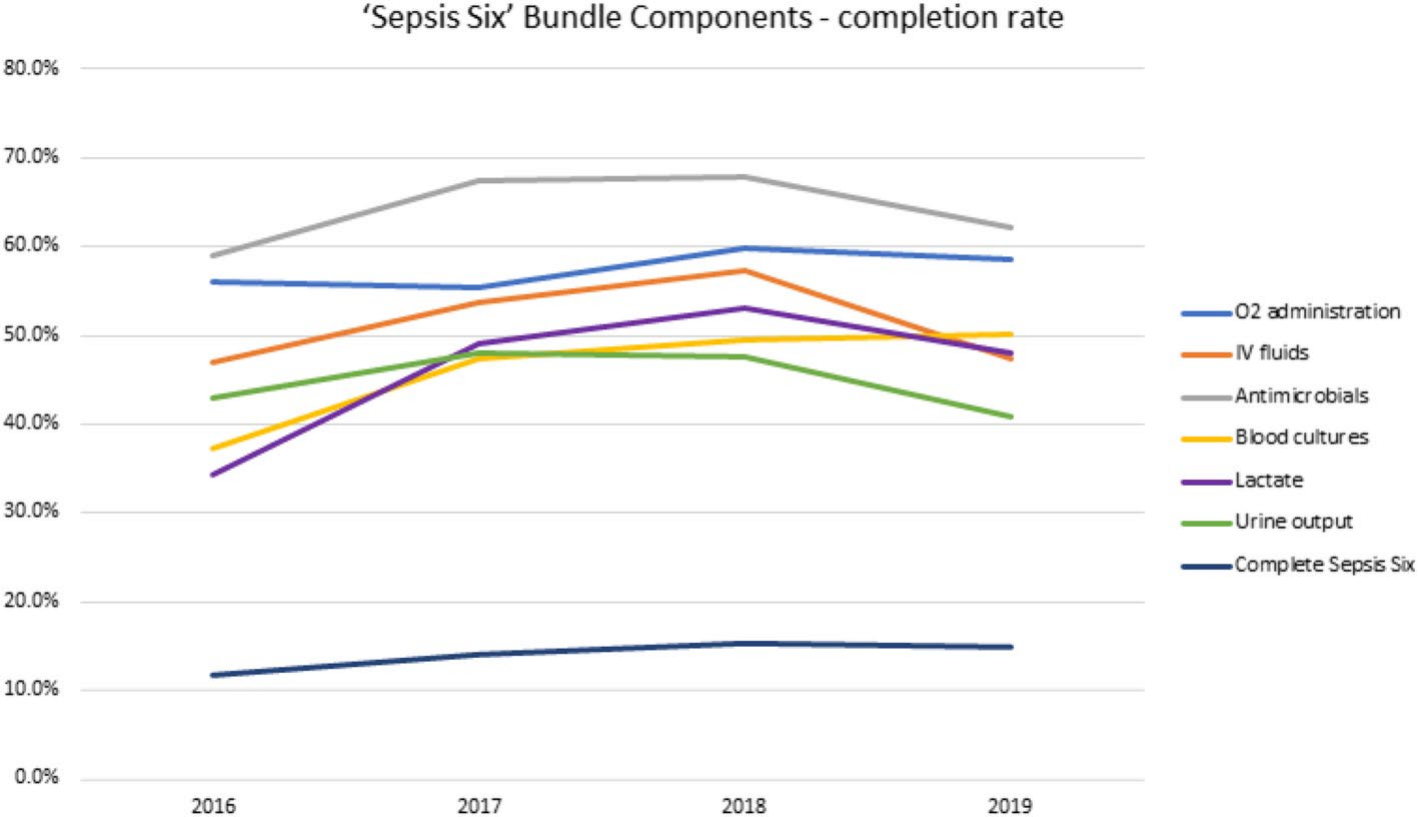
‘Sepsis Six’ bundle completion rates during the study period. Data is presented for overall (dark blue line) and individual bundle elements: O_2_ administration (blue line), IV fluids (orange line), antimicrobials (grey line), blood cultures (yellow line), lactate (purple line), urine output measurement (green line).

The completion of overall, as well as individual elements of the ‘Sepsis Six’ bundle over time is further presented in Fig. 2.

**Figure 3:**
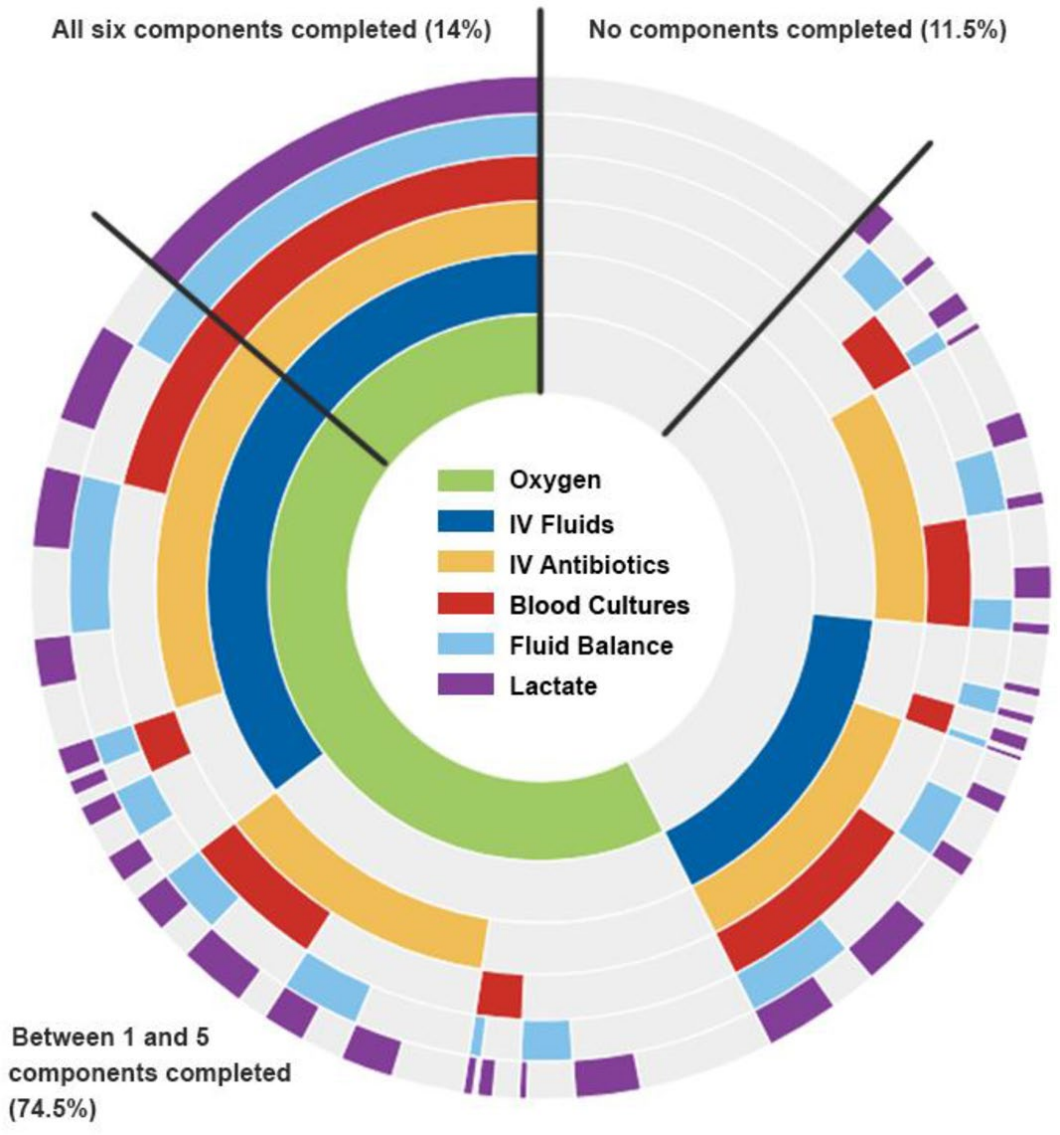
'Sepsis Six’ bundle element completion rates. A sunburst plot illustrating the frequency of completion of each component of the Sepsis Six bundle for the total events from 2016 to 2019 (n = 1588, with missing values removed). The coloured areas denote the Sepsis Six component has been completed, the grey areas denote where a component has not been completed. Working from the center, the frequency of each combination of Sepsis Six bundle components is illustrated. Plot created using R software (Version 1.2.1335), utilising packages ggplot (v 3.3.3) and sunburstR (v2.1.5)^24,25^. IV: intravenous.

When examined individual bundle elements, lactate measurement and obtaining blood cultures improved over time; however all elements were completed well below 70% of occasions (Fig. 2). We found no differences between organisations in completing ‘Sepsis Six’ bundles (as displayed in Supplementary Figure 2). Regardless of the number of bundle elements completed, we did not find any difference in the mortality across the years (Supplementary Figure 3). No discernible trends or patterns were identified when we examined the completion of individual and combined bundle elements (Fig. 3 and further interactive visualisation in Supplementary Figure 4 plus summary of most frequent combinations shown in Supplementary Figure 5) or when this was plotted against the patients’ NEWS across the study period (demonstrated in Supplementary Figure 6).

Blood cultures were obtained from 632 (46.0%) patients, of which 89 (14.1%) were positive for growth. Sputum sampling had a substantially higher positivity rate (35.9%). Other microbiology samples were infrequently collected (Table 3).

**Table 3:** Sepsis management—culture collection.

Specimen	Collected (n = 1651)	Positive culture
Blood	632 (46.0%)	89 (14.1%)
Sputum	170 (13.9%)	61 (35.9%)
Urine	455 (33.4%)	86 (18.9%)
Wound	112 (8.2%)	54 (48.2%)
CSF	8 (0.6%)	0 (0%)

CSF Cerebrospinal fluid.

Antimicrobials were administered to 743 (64.3%) patients. Piperacillin-tazobactam, followed by co-amoxiclav and clarithromycin were the commonly used antibiotics used over the four-year period and are illustrated in Supplementary Figure 7.

One hundred and seventy (10.7%) patients were seen by critical care outreach; the ‘Sepsis Six’ bundle was completed significantly more often in this group (54/170, 32.0%) than for patients who were not reviewed by critical care outreach (168/1385, 11.6%; *p* < 0.0001). However, when plotted as a patient pathway these effects became less pronounced (illustrated in the river-plot in Supplementary Figure 8).

In planned sensitivity analysis we found that the percentage number of patients with NEWS 6 or above (overall n = 486, 29.4%) did not change significantly over the study period (Table 1). In this group, more patients had a ceiling of care (such as ward level care only or not for intubation decision) and also DNA-CPR orders in place (19.7% vs 9.5%, *p* < 0.0001 and 37.8% vs 21.2%, *p* < 0.0001, respectively) compared to the less acutely unwell population. The completion of the ‘Sepsis Six’ bundle was significantly higher for patients with NEWS 6 or above (20.9% vs 11.1%, *p* < 0.0001) but unchanged over the study period, as was the completion rate for individual bundle elements (shown in Supplementary Figure 9).

### Survival analysis

**Figure 4:**
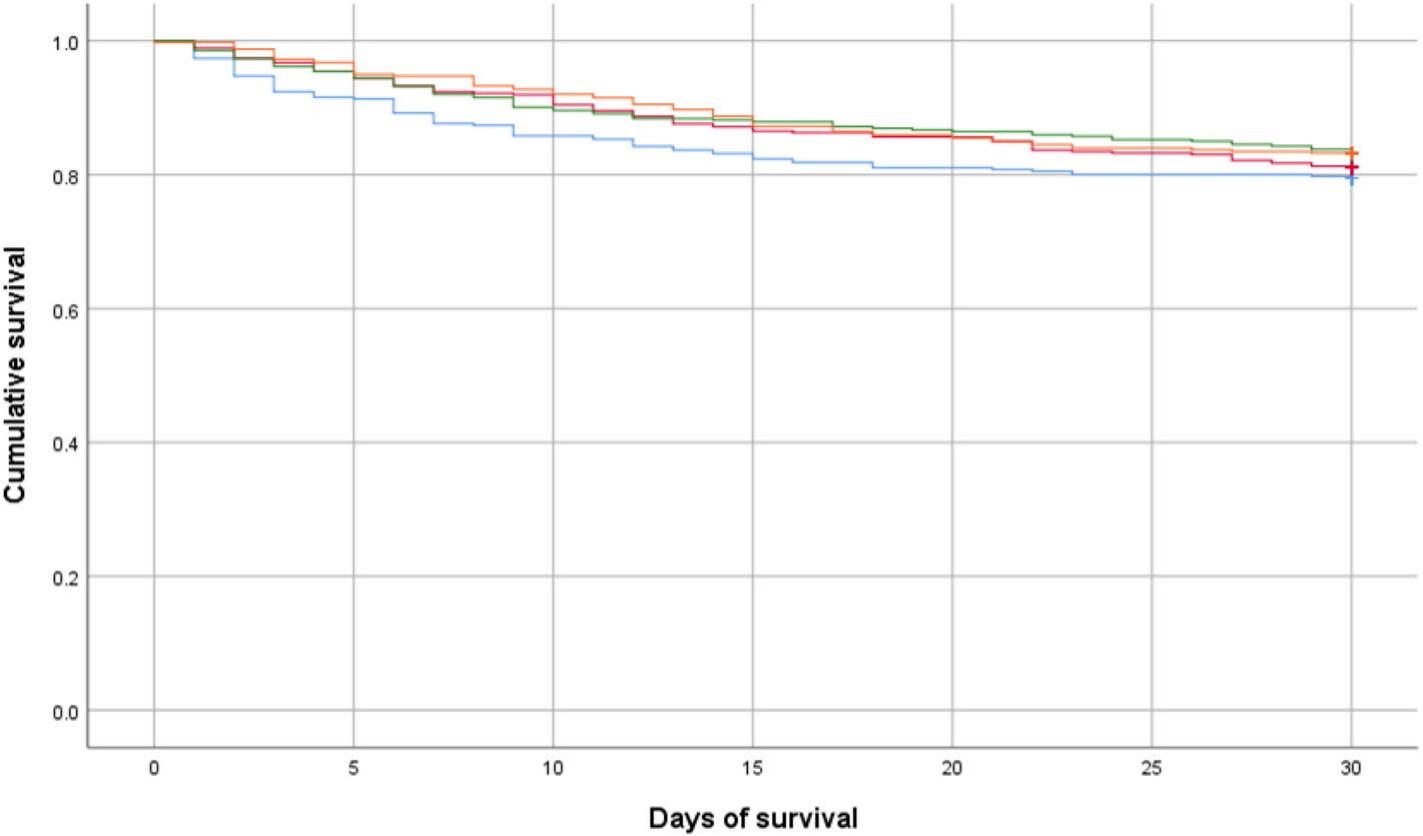
Survival difference of patients with sepsis presenting to emergency department or general wards in fourteen Welsh hospitals in the years; 2016 (blue line), 2017 (red line), 2018 (green line) and 2019 (orange line), *p* = 0.39.

Overall, 1349 of 1651 patients (81.7%) survived to 30 days with a mean survival time of 26.5 days (95% CI 26.1–26.9). We found no difference in patient survival at 30 days between each year of study (Table 1 and Fig. 4).

We observed significantly higher mortality in patients with NEWS 6 or above (23.5% vs 16.1%, *p* < 0.0001). Overall 90-day survival for years 2017 – 2019 was 74.7% (949/1271). There was no difference in patient survival at 90 days between each year (see Kaplan–Meier curve in Supplementary Figure 10).

### Risk factors of mortality

On multivariate regression analysis, we identified older age, heart failure, recent chemotherapy, higher frailty score and do not attempt cardiopulmonary resuscitation (DNA-CPR) orders as significantly associated with increased mortality in patients with sepsis (Table 4).

**Table 4:** Multivariate Cox regression analysis of the risk factors for mortality in sepsis patients. Values are Hazards Ratio (95%CI).

Variables	Hazards ratio (95% CI)	*P* value
**Demographics**		
Age	1.04 (1.031.05)	< 0.0001
Male	1.30 (0.96–1.74)	0.09
**Co-morbidities**		
COPD	0.95 (0.70–1.30)	0.77
Diabetes	0.81 (0.55–1.18)	0.26
Drugs of abuse	0.46 (0.06–3.37)	0.45
HF	1.50 (1.03–2.20)	0.04
HTN	1.08 (0.80–1.46)	0.61
IHD	0.87 (0.60–1.27)	0.48
Liver disease	1.07 (0.49–2.32)	0.86
Neuromuscular	1.33 (0.61–2.89)	0.47
Recent chemotherapy	3.12 (1.86–5.21)	< 0.0001
Frailty score	1.17 (1.05–1.30)	< 0.01
DNA-CPR	1.47 (1.03–2.09)	0.03
NEWS ≥ 6	0.84 (0.59–1.20)	0.34
**Management**		
Complete sepsis six bundle	0.67 (0.42–1.08)	0.10
All Wales screening tool	0.86 (0.58–1.29)	0.48
Seen by critical care outreach	1.13 (0.72–1.77)	0.60

COPD chronic obstructive pulmonary disease, HF heart failure, HTN hypertension, IHD ischemic heart disease, DNA-CPR do not attempt cardiopulmonary resuscitation order. NEWS National Early Warning Score.

## Discussion

We identified that sepsis management in Wales (according to sepsis screening tool application and ‘Sepsis Six’ bundle compliance) has not altered over the four-year study period and that mortality remain largely unchanged.

We found the demographic of the study population remained the same for each year, consisting of predominately frail and elderly patients with significant comorbidities. Approximately a third of the patients had a high NEWS and this group had higher likelihood of care limitations and DNA-CPR orders in place. Over the study period, there was no change in the short or medium-term mortality in the cohort, with approximately three out of four patients alive at 90 days. Our data opposes beliefs expressed that within the last decade the implementation of resuscitation bundles has led to better recognition of sepsis, in turn increasing the reported incidence of sepsis and reducing its apparent mortality^6,26,27^. Our observations are supported by recent analysis of studies identifying sepsis using direct clinical indicators of infection and organ dysfunction, suggesting that over the last decade the incidence and mortality of sepsis has in fact remained stable^28,29^.

Our findings that older age and higher frailty score are both associated with increased risk of mortality from sepsis, within an elderly population with high comorbidity burden, emphasise the threat of sepsis to patients throughout our hospitals^3,30–32^. The observations that heart failure and previous chemotherapy are associated with higher mortality from sepsis, are not new and are supported by results from large international cohorts^11,33,34^.

‘Sepsis Six’ bundle completion remained low with a mean of 14.0% over four years. The lack of improvement in completion of bundles probably underlines the significant problem of sepsis recognition outside of the ICU^35^. Our results support previously published UK and international data and highlight a significant concern in the real-world operationalising of response, which show significantly lower compliance in comparison to the sepsis performance measure (SEP-1) initiative or the resuscitation bundle promoted by the SCC^13,36^. Alarmingly, only one in five patients received the full bundle in a group with higher risk of deterioration, i.e. NEWS 6 or above, whilst only one out of ten patients received the full bundle in the lower acuity group, with no change over the four years. However, we found that patients who were reviewed by critical care outreach were more often treated with the full ‘Sepsis Six’ bundle and had antibiotics administered. This result is in line with previous experiences, where introduction of a dedicated team has improved compliance with the bundle^37^. Whilst our study did not find any association between critical care outreach involvement and mortality, it is possible that illness severity is a confounding factor here. It’s important to note that critical care outreach provision was variable across Wales during the study period^38^. Not every organisation had these services available and none of the hospitals had 24/7 critical care outreach on site. Furthermore, the existing critical care outreach services were nurse-led and delivered and at the time of the study, they did not have appropriate privileges for drug prescription and in some cases ordering tests either. Taking this into account, the associated three-fold increase in the ‘Sepsis Six’ bundle completion is remarkable.

Our results point towards system failure to respond to sepsis as a medical emergency and highlight the need for policy change in the Welsh NHS in response to sepsis. Despite the introduction of the quality improvement target for ‘Sepsis Six’ bundle completion in 2017/2018 by the Welsh Government, we have seen little change across the study years^17^. This quality improvement target was not accompanied by financial incentives or any additional funding. Importantly, there is no publicly available report about the baseline measured by this methodology and any potential improvement attained since 2017/2018 in the Welsh NHS organisations. The implementation of care bundles have been shown to have significant institutional barriers, which may not be overcome by traditional plan-do-study-act quality improvement cycles^39,40^. Importantly, neither the ‘Sepsis Six’ bundle, nor the SEP-1 bundle has been tested in a robust randomised controlled trial (RCT) and their perceived effectiveness has been derived from observational before and after studies with high risk of bias^41^. We believe, based on the individual bundle element compliance figures, that our data may show the presence of clinical equipoise for an RCT to test whether a bundle approach indeed improves outcomes compared to the current apparent standard care of administering supplemental oxygen and antibiotics to the majority of the patients with NEWS above 6. RECOVERY and REMAP-CAP have demonstrated the potential efficiency and effectiveness of adaptive platform trials^42–44^, and the recently funded Sepsis Trials in Critical Care (SEPTIC) platform (NIHR 17/136/02) illustrates such an approach in sepsis management. Adaptive platform trials create opportunities for ‘learning health care systems’ which promote efficient knowledge generation and transfer, use simple and purposeful data systems with transparent quality metrics, and integrate these into clinical, academic and commissioning structures^45–47^. Considering the significant evidence gap in the ward based sepsis care demonstrated in our study, we propose that a similar platform trial is necessary to delineate which timely, ward-based interventions can reduce mortality in patients with sepsis at the highest risk of adverse outcomes^46,48^.

There are certain limitations to our study. Firstly, the dataset was designed to enable a sufficiently comprehensive list of clinical and laboratory parameters while being small enough to maintain data reliability. Data collection was performed by medical students at different stages of training, introducing potential bias. To counter this, robust online and in-person training was cascaded, and we ensured that medical student hospital leads in subsequent years had participated as data collectors^16,20,21^. We also maintained the core clinical leadership of the group throughout the study. Secondly, we have only collected longer-term outcome data and cause of death on a subset of patients and our long-term follow-up data is yet to be linked with the Welsh Secure Anonymous Information Linkage (SAIL) databank^31,49^. The true human cost of sepsis in terms of re-hospitalisation and patient reported outcomes cannot be estimated from our results. Thirdly, although one of the largest in-depth sepsis studies in the UK, the sample-size is relatively small. However, we could not see any differences in sepsis incidence or outcomes based on geographical area, hospital status or size and we ensured that all acute hospitals in Wales participated in each year of the study^16,20,21^. Lastly, the point-prevalence design might have led to a systematic underestimate of compliance with ‘Sepsis Six’ completion; however, despite being mandated in the NHS Wales Delivery Framework in 2017^17^, there is no publicly available data generated by Welsh Health Boards to provide a comparison on longer-term longitudinal changes of this quality improvement index. Moreover, engagement of participating hospitals with our point-prevalence study has remained high and our results have been consistent across the study period.

## Conclusions

In summary, our data suggests that despite efforts to increase sepsis awareness within the NHS, there is poor compliance with the sepsis care bundles and there has been no change in outcomes over the study period. Our results highlight the ongoing need for clinical trials to determine which time-sensitive ward-based interventions are most likely to reduce mortality in patients with highest risk of death and which should be adopted by learning healthcare systems.

## Supplementary Information

Supplementary Legends.

Supplementary Figure 1.

Supplementary Figure 2.

Supplementary Figure 3.

Supplementary Figure 4.

Supplementary Figure 5.

Supplementary Figure 6.

Supplementary Figure 7.

Supplementary Figure 8.

Supplementary Figure 9.

Supplementary Figure 10.

## Data Availability

The datasets used and/or analysed during the current study are available from the corresponding author on reasonable request.
