# Supplementary material for: Four consecutive yearly point-prevalence studies in Wales indicate lack of improvement in sepsis care on the wards
Source: Sci Rep. 2021 Aug 10;11:16222. doi: 10.1038/s41598-021-95648-6 (PMC8355110; doi:10.1038/s41598-021-95648-6)
Supplement: Supplementary file 1 — Supplementary Legends. [file 41598_2021_95648_MOESM1_ESM.docx]

**Four consecutive yearly point-prevalence studies in Wales indicate lack of improvement in sepsis care on the wards**

**Captions for Supplementary Figures**

**Supplementary Figure 1.** PHW_Sepsis_Screening_Leflet_11.pdf

All Wales Sepsis/Severe sepsis screening tool

**Supplementary Figure 2.** CC Supplementary Figure 2.tiff

Proportion of cases with completed ‘Sepsis Six’ bundle per University Health Board (UHB).

The number of and percentage of patients where Sepsis Six bundle was completed is shown of the Y axis, grouped by the participating Health Boards on the y axis. ABM: Abertawe Bro Morganwgg

**Supplementary Figure 3.** CC Supplementary Figure 3 Sepsis6 bundle per year V2.tiff

Sepsis Six bundle element completion and mortality in each year

Percentage of Sepsis Six bundle components completed per year. The number of Sepsis Six bundle components completed is shown of the x axis, with the frequency (%) of each bundle component group per year shown on the y axis. Survival at 30 days from the event is shown by colour.

**Supplementary Figure 4.** CC Supplementary Figure 4 – Interactive plot.html

Sepsis bundle element completion

A sunburst plot illustrating the frequency of completion of each component of the Sepsis Six bundle for the total events from 2016-2019 (n = 1588, with missing values removed). The coloured areas denote the Sepsis Six component has been completed, the grey areas denote where a component has not been completed. Working from the centre, the frequency of each combination of Sepsis Six bundle components is illustrated. IV: intravenous.

**Supplementary Figure 5.** CC Supplementary Figure 5 upset_plot_perc.png

Completion of the most frequent Sepsis Six bundle elements

Frequency of each Sepsis Six bundle combination for the total events from 2016-2019. Only the 15 most common bundle combinations are shown, ordered by frequency, out of a total 64 different combinations recorded. The x axis shows possible Sepsis Six bundle combinations. Each filled-in node shows a component is present in the bundle, with the vertical lines linking each relevant component within the combination [1,2]. The frequency (%) of each combination is shown along the upper y axis. IV: intravenous

References

1. Gu Z, Eils R, Schlesner M. Complex heatmaps reveal patterns and correlations in multidimensional genomic data. Bioinformatics. 2016;32:2847-9.
2. Conway JR, Lex A, Gehlenborg N. UpSetR: an R package for the visualization of intersecting sets and their properties. Bioinformatics. 2017;33:2938-40.

**Supplementary Figure 6.** CC Supplementary Figure 6 Sepsis 6 and NEWS V2.tiff

Sepsis Six bundle completion by total NEWS.

The number of Sepsis Six bundle components completed is shown on the x axis, with the associated total NEWS at the time of the event shown by the y axis. Each point represents a single patient. Points are coloured to represent year. NEWS: National Early Warning Score.

**Supplementary Figure 7.** CC Supplementary Figure 7.tif

Antibiotics use by year

The number of patients who had the different antibiotics administered is shown on the y axis.

**Supplementary Figure 8.** CC Supplementary Figure 8.png

Patient pathway analysis

Width of bands depict proportions of the population

**Supplementary Figure 9.** new Supplementary Figure 9.tif

‘Sepsis Six’ bundle completion rates during the study period for patients with NEWS 6 or above.

Data is presented for overall (dark blue line) and individual bundle elements: O_2_ administration (blue line), IV fluids (orange line), antimicrobials (grey line), blood cultures (yellow line), lactate (purple line), urine output measurement (green line).

**Supplementary Figure 10.** new Supplementary Figure 10.tif

90-day survival

Survival difference of patients with sepsis presenting to emergency department or general wards in fourteen Welsh hospitals in the years; 2017 (blue line), 2018 (red line) 2019 (green line).
