# Supplementary material for: Four consecutive yearly point-prevalence studies in Wales indicate lack of improvement in sepsis care on the wards
Source: Sci Rep. 2021 Aug 10;11:16222. doi: 10.1038/s41598-021-95648-6 (PMC8355110; doi:10.1038/s41598-021-95648-6)
Supplement: Supplementary file 2 — Supplementary Figure 1. [file 41598_2021_95648_MOESM2_ESM.pdf]

Sepsis/Severe Sepsis
Screening Tool

Are any two of the following SSI criteria present?

- Temperature <36 or >38.3⁰C
- Heart rate >90bpm
- WCC >12 or <4 x10⁹/l
- Respiratory rate >20/min
- Acutely altered mental state
- Hyperglycaemia in the absence of diabetes

If yes, patient has SSI

Does your patient have a history or signs suggestive of a new infection? For example:

- Cough/ sputum/ chest pain
- Abdo pain/ distension/ diarrhoea
- Line infection
- Endocarditis
- Dysuria
- Headache with neck stiffness
- Cellulitis/ wound infection/ septic arthritis

If yes, patient has SEPSIS

Any signs of organ dysfunction?

- SBP < 90mmHg or MAP < 65mmHg
- Urine output < 0.5ml/kg/hr for 2 hrs
- INR > 1.5 or aPTT > 60s
- Bilirubin > 34μmol/l
- Lactate > 2mmol/l
- New need for oxygen to keep SpO2 >90%
- Platelets < 100 x 10 ⁹/1
- Creatinine > 177 mmol/l

If no, treat for **SEPSIS**:

- Oxygen
- Blood cultures
- IV antibiotics
- Fluid therapy
- Reassess for SEVERE SEPSIS with hourly observations

If yes, patient has **SEVERE SEPSIS**
Start SEVERE SEPIS CARE PATHWAY

Survive SEPSIS **www.survivesepsis.org**
Ron Daniels October 2007 updated 2008 C&V

1000 LIVES O FYWYDAU

Document to be kept in patient's notes

Patient name: **PID:**

Date: **Ward:**

Severe Sepsis Care Pathway – First Hour Care Duties

Yes **Could this patient have sepsis?** No

Yes **Apply Severe Sepsis Screening Tool**

Negative **Reassess patient** Apply appropriate Management plan

Sepsis Six©	Time	Initial	Reason not done or result
**1. Oxygen:** high flow 15l/min via non-rebreathe mask. Target saturations > 94%			
**2. Blood cultures:** take at least one set plus all relevant blood tests eg FBC, U&E, LFT, clotting, glucose. *Consider urine/ sputum/ swab samples.*			
**3. IV antibiotics** as per trust guidelines			
**4. Fluid resuscitate:** if hypotensive give boluses of 0.9% saline or Hartmann's 20 ml/kg up to a max of 60ml/kg			
**5. Serum lactate** and Hb: ABG Ensure Hb > 7g/dl			
**6. Catheterise** and commence fluid balance			

Plus

Referral to Critical Care.

Do you need to discuss with your consultant – on-call first?

Please think before referring is this episode reversible? Have all the above been completed and the patient reviewed within one hour and a PMH/Co-morbidity history taken?

One hour time check: all steps done? Yes ☐ No ☐

Name: Signature:

Designation: Bleep No.:

Patient name: **PID:**

Date: **Ward:**

6 Hour Resuscitation Bundle (assisted care)

Systolic BP **<90mmHg** or MAP **<65mmHg** or a fall of >40mmHg from baseline

Yes No

Septic shock present!

Confirm first hour care duties complete
Apply Early Goal Directed Therapy

Lactate>4mmol/l?

Yes No

Severe sepsis, no shock

Ensure management plan is documented in notes

Ensure hourly obs taken, recorded and acted upon.

REASSESS frequently!

	Time achieved	Initial	Reason not done or result
**1.** Ensure patient has received adequate **fluid resuscitation**: boluses of 20ml/kg 0.9% saline or Hartmann's to a max of 60ml/kg			
**2.** If still shocked *(low BP/ low urine output/ high lactate)* Ensure **Critical Care** attend urgently			
**3.** If still shocked *(low BP/ low urine output/ high lactate)* **insert central venous catheter** under USS guidance (only if competent; otherwise seek help)			
4. Aim to achieve **CVP 8-12mmHg** with Care,Check CVP Monitor			
**5.** Take heparinised sample from central line (use ABG syringe): check **ScvO2>70%**			
**6.** Ensure **Hb>7g/dl**: consider transfusion if necessary			
**7.** Consider **noradrenaline** if still shocked or **dobutamine** if ScvO2 < 70%			

6 hour time check: All steps complete? Yes ☐ No ☐

Name: Signature:

Designation: Bleep/ID Card No.:

SBAR Reporting

**S**	Date: .... Time: ......:....................... (24hrs)<br>Drs name: ....<br>My name is ....<br>From Ward/Dept ....<br>I am calling about (patient name) ....<br>The problem is ....
**B**	The patient was admitted with ....<br>on ....../....../.....<br>Relevant PMH ....<br>Resuscitation status ....
**A**	The patient has a PAR score of ....<br>Airway ....<br>Breathing ....<br>Circulation ....<br>Disability ....<br>Exposure ....<br>Other relevant factors e.g. Sepsis screening, blood results, pain, urine output<br>..
**R**	I request you review the patient within the next .... hrs/mins<br>(enter agreed timescale e.g. 30mins)<br>Document any initial instructions<br>..<br>..<br>Patient reviewed by Dr at ......:...... (24hrs)
