# Supplementary figures and images for: Four consecutive yearly point-prevalence studies in Wales indicate lack of improvement in sepsis care on the wards
Source: Sci Rep. 2021 Aug 10;11:16222. doi: 10.1038/s41598-021-95648-6 (PMC8355110; doi:10.1038/s41598-021-95648-6)

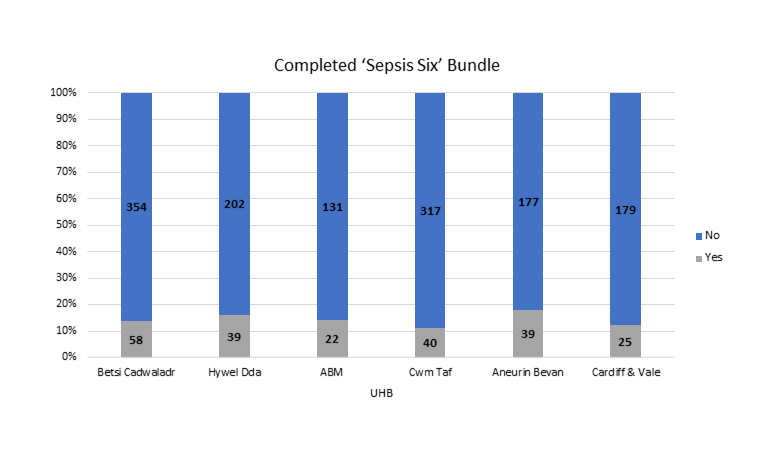

Supplement: Supplementary file 3 — Supplementary Figure 2. [file 41598_2021_95648_MOESM3_ESM.tiff]

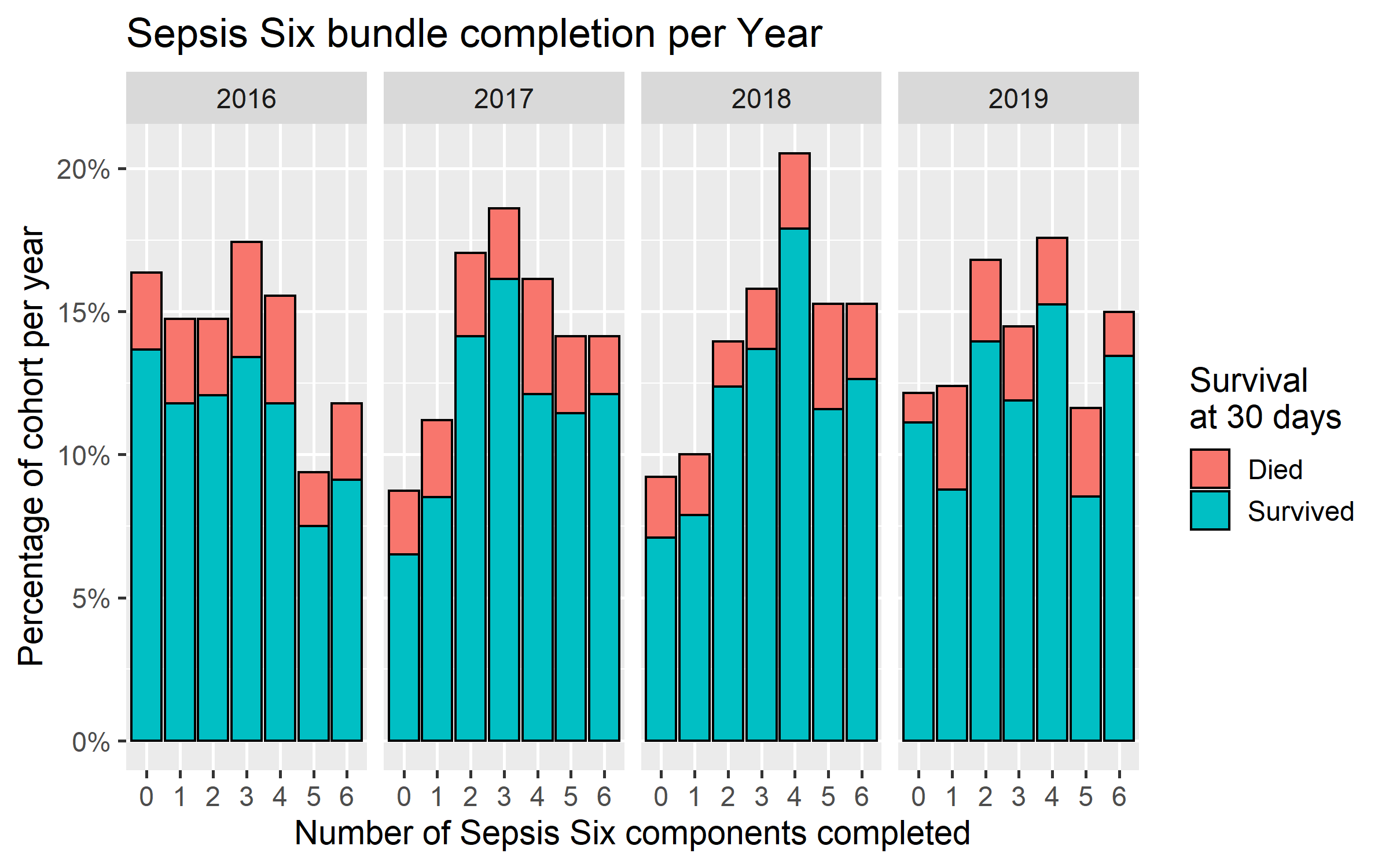

Supplement: Supplementary file 4 — Supplementary Figure 3. [file 41598_2021_95648_MOESM4_ESM.tiff]

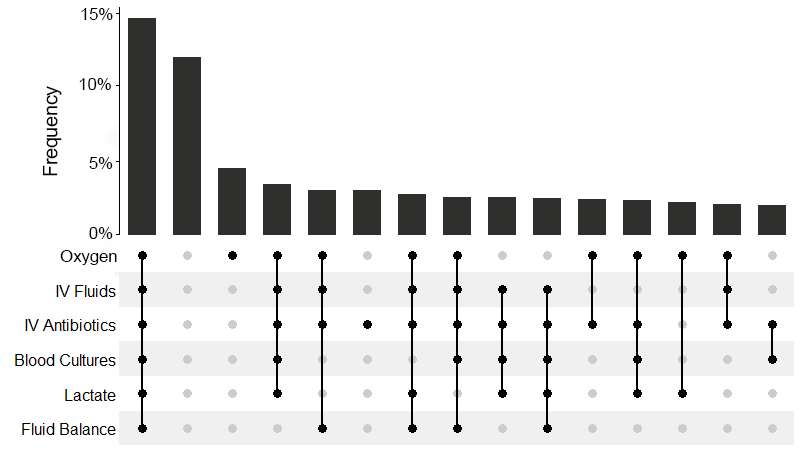

Supplement: Supplementary file 6 — Supplementary Figure 5. [file 41598_2021_95648_MOESM6_ESM.png]

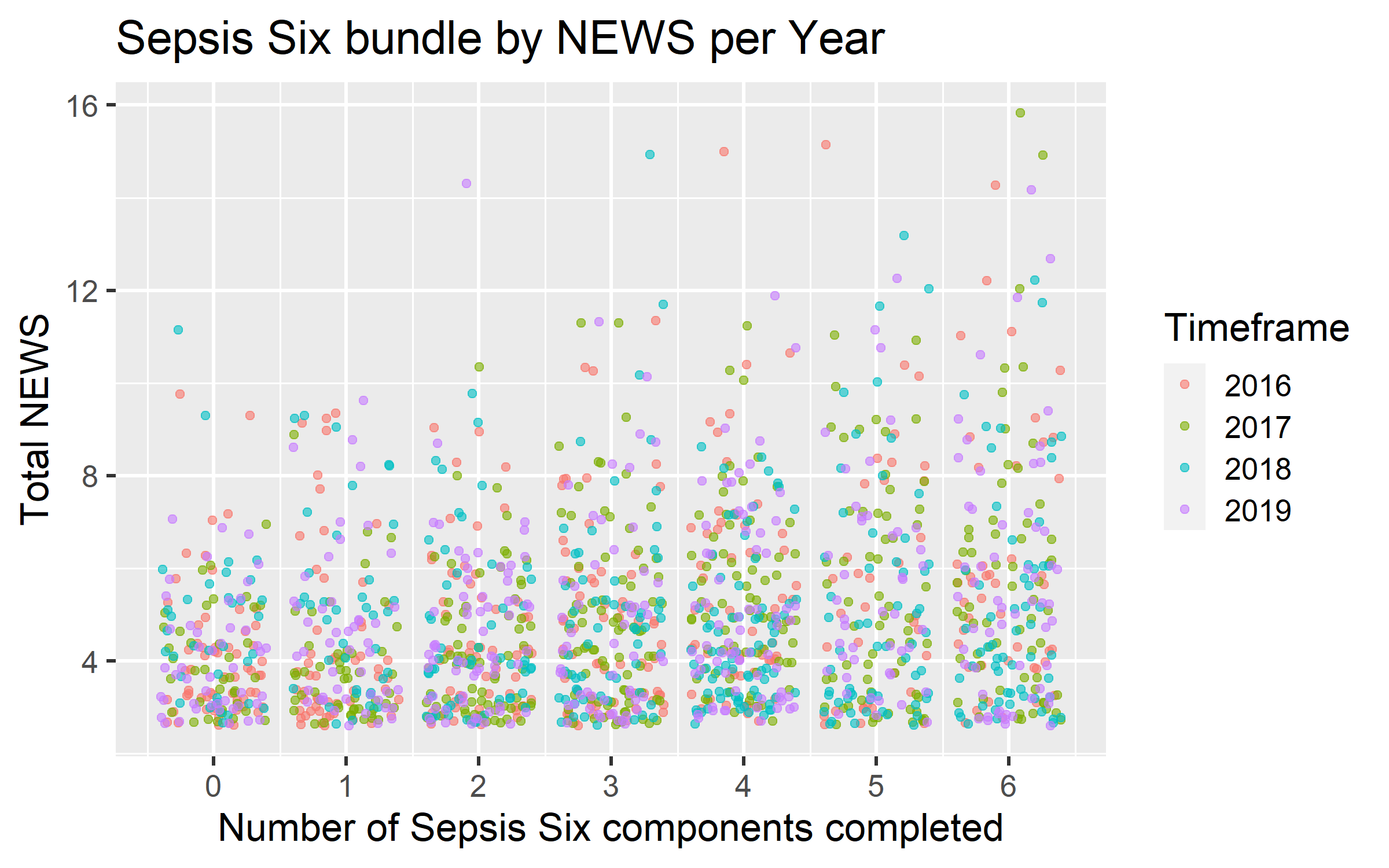

Supplement: Supplementary file 7 — Supplementary Figure 6. [file 41598_2021_95648_MOESM7_ESM.tiff]

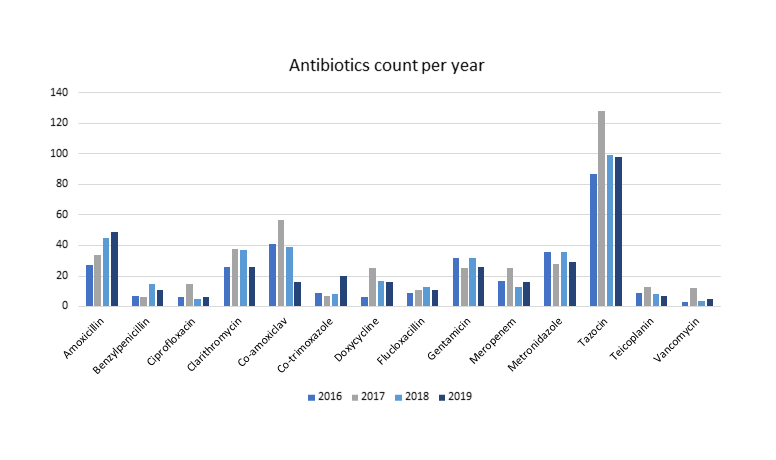

Supplement: Supplementary file 8 — Supplementary Figure 7. [file 41598_2021_95648_MOESM8_ESM.tif]

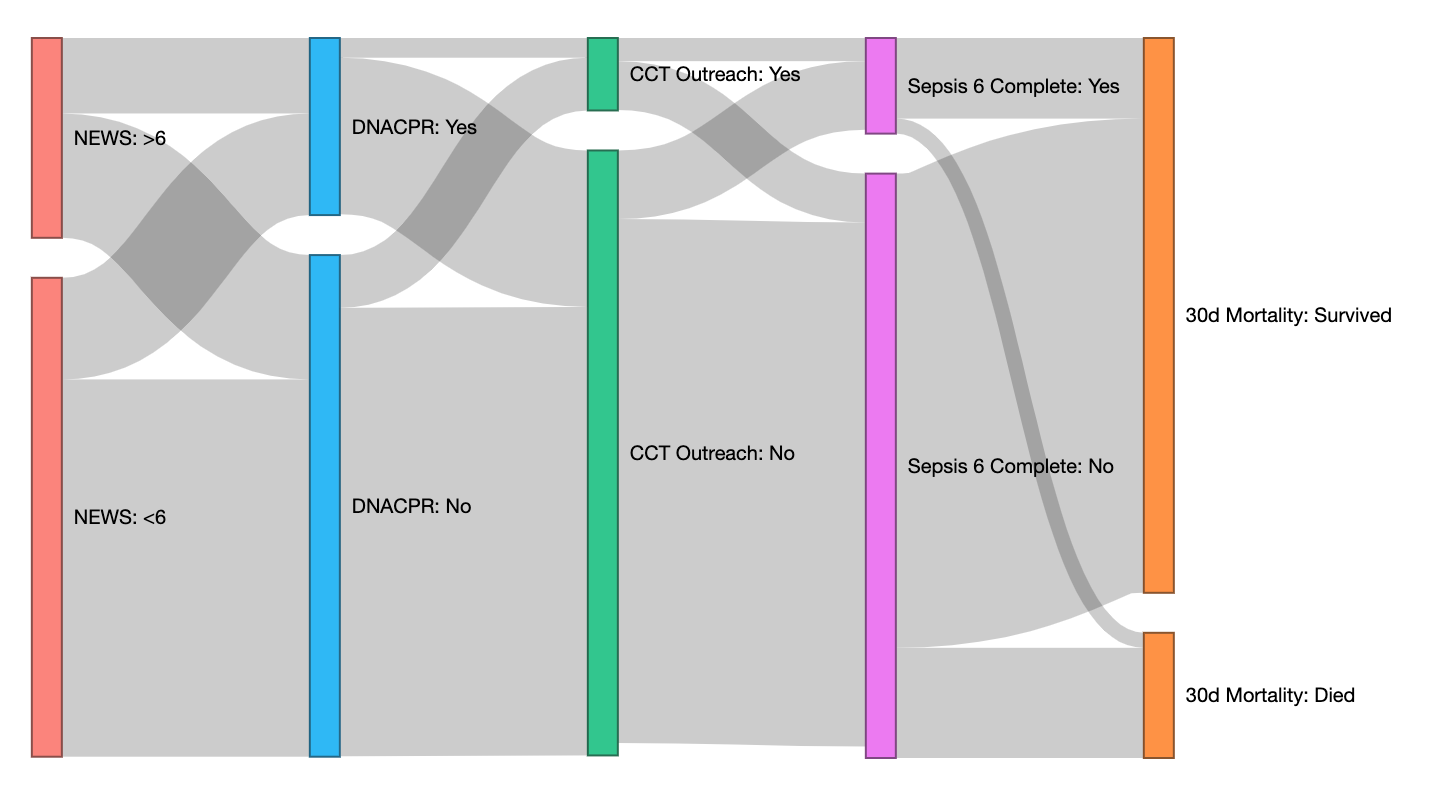

Supplement: Supplementary file 9 — Supplementary Figure 8. [file 41598_2021_95648_MOESM9_ESM.png]

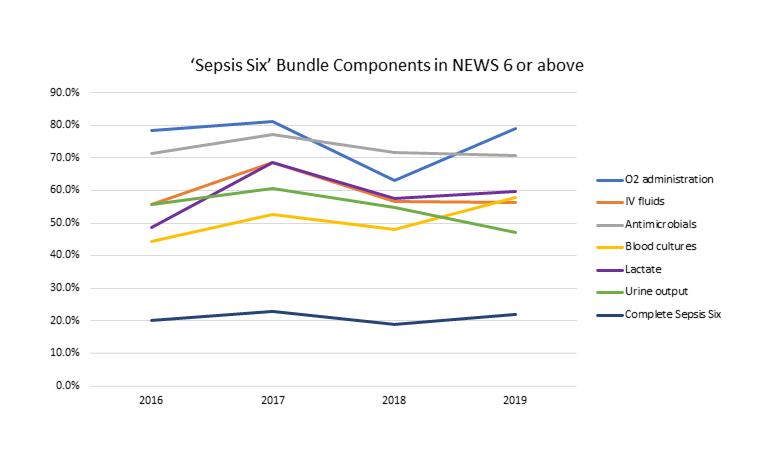

Supplement: Supplementary file 10 — Supplementary Figure 9. [file 41598_2021_95648_MOESM10_ESM.tif]

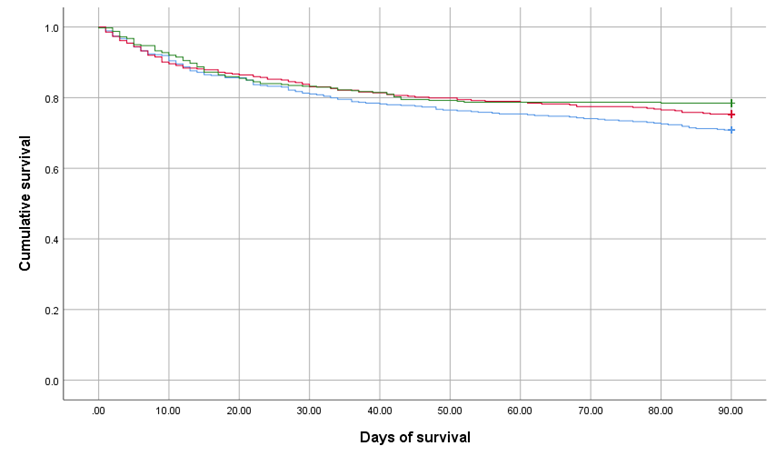

Supplement: Supplementary file 11 — Supplementary Figure 10. [file 41598_2021_95648_MOESM11_ESM.tif]
