# Supplementary material for: Four consecutive yearly point-prevalence studies in Wales indicate lack of improvement in sepsis care on the wards
Source: Sci Rep. 2021 Aug 10;11:16222. doi: 10.1038/s41598-021-95648-6 (PMC8355110; doi:10.1038/s41598-021-95648-6)
Supplement: Supplementary file 5 — Supplementary Figure 4. [file 41598_2021_95648_MOESM5_ESM.html]

Supplementary Figure - Interactive Plot


# Supplementary Figure - Interactive Plot

#### Laura Tan

#### 12/03/2021

---

**Lack of improvement in sepsis care on the wards: results of four consecutive yearly point-prevalence studies in Wales**

Welsh Digital Data Collection Platform collaborators. wddcp@gmail.com @wddcp

Correspondence to: Prof T. Szakmany, Department of Anaesthesia, Intensive Care and Pain Medicine, Division of Population Medicine, Cardiff University, Cardiff, UK  
Email: SzakmanyT1@cardiff.ac.uk @iamyourgasman

---

  Supplementary Figure: Interactive Sunburst Plot HTML

Legend

**Legend**  
An interactive sunburst plot illustrating the frequency of completion of each component of the Sepsis Six bundle for the total events from 2016-2019 (n = 1588, with missing values removed). The coloured areas denote the Sepsis Six component has been completed, the grey areas denote where a component has not been completed. Working from the center, the frequency of each combination of Sepsis Six bundle components is illustrated and can be hovered over with the mouse to view the full breakdown. IV: intravenous.
